# Does Vehicle-2-X Radio Transmission Technology Need to Be Considered within Accident Analysis in the Future?

**DOI:** 10.3390/s22249832

**Published:** 2022-12-14

**Authors:** Maximilian Bauder, Tibor Kubjatko, Thomas Helmer, Hans-Georg Schweiger

**Affiliations:** 1 Technische Hochschule Ingolstadt, CARISSMA Institute of Safety in Future Mobility, Esplanade 10, 85049 Ingolstadt, Germany; 2 Institute of Forensic Research and Education, University of Zilina, 010 26 Žilina, Slovakia; 3 Technische Hochschule Ingolstadt, CARISSMA Institute of Electric, Connected, and Secure Mobility, Esplanade 10, 85049 Ingolstadt, Germany

**Keywords:** cooperative intelligent transportation systems, C-ITS, C-V2X, LTE-V2X, DSRC, IEEE 802.11p, accident scenarios, accident analysis, accident reconstruction

## Abstract

In this analysis, Cooperative Intelligent Transportation System relevant scenarios are created to investigate the need to differentiate Vehicle-to-X transmission technologies on behalf of accident analysis. For each scenario, the distances between the vehicles are calculated 5 s before the crash. Studies on the difference between Dedicated Short-Range Communication (IEEE 802.11p) and Cellular Vehicle-to-X communication (LTE-V2C PC5 Mode 4) are then used to assess whether both technologies have a reliable connection over the relevant distance. If this is the case, the transmission technology is of secondary importance for future investigations on Vehicle-to-X communication in combination with accident analysis. The results show that studies on freeways and rural roads can be carried out independently of the transmission technology and other boundary conditions (speed, traffic density, non-line of sight/line of sight). The situation is different for studies in urban areas, where both technologies may not have a sufficiently reliable connection range depending on the traffic density.

## 1. Introduction

In the field of cooperative intelligent transportation systems (C-ITS), two different radio transmission technologies are currently competing, which do not interoperate due to different processing and modulation methods [1]. IEEE 802.11p represents a WLAN-based technical implementation within Dedicated Short-Range Communication (DSRC), which is known in Europe as ITS-G5 and was the first to be standardised for Vehicle-to-X communication (V2X) [2]. On the other hand, a cellular transmission technology with sidelink (C-V2X) was developed and standardised based on Long Term Evolution (LTE) mobile radio technology [1,3]. Both technologies have pros and cons, which this paper will not discuss further. Instead, this paper aims to discuss the necessity of differentiating the transmission technology for future studies on C-ITS in the field of accident analysis, which has not yet been investigated to the best of our knowledge.

The discourse on the two transmission technologies is currently a highly controversial topic within the V2X community, evidenced by many publications. The performance has been investigated using simulations at the link level [4,5,6], simulations at the system level [7,8,9,10,11,12] and field tests [13,14,15]. The development and standardisation in this field are also highly dynamic. For example, further development of IEEE 802.11p to IEEE 802.11bd is currently being worked on to increase the range and data throughput [3]. At the same time, C-V2X is being further developed into New Radio V2X (NR-V2X) in the course of 5G technology [3,16].

In the field of accident analysis, however, the topic of V2X has not yet been addressed, as shown in [17]. The analysis in [17] revealed that it should be investigated whether the radio transmission technology needs to be differentiated for future research in the field of accident analysis. This is particularly important for conducting further studies in the field of accident analysis on V2X. It is currently not certain which technology will prevail, and future studies with the “wrong” transmission technology could become insignificant. At the same time, conducting the studies with both technologies represents an uneconomical additional expense.

This work aims to investigate the necessity of differentiating between the two transmission technologies in the context of accident analysis. To answer this question, a novel approach is suggested in which relevant scenarios were developed and investigated with worst-case conditions for the reliable transmission range. In this approach, the distances of the communicating vehicles 5 s before the crash are calculated and compared with the reliable range from the existing literature. Suppose the reliable range of both technologies is greater than the vehicles’ distance from each other; in that case, the transmission technology does not matter from the point of view of the accident analysis, since both vehicles were reliably in contact and could exchange messages throughout the entire accident sequence.

## 2. Materials and Methods

**Figure 1:**
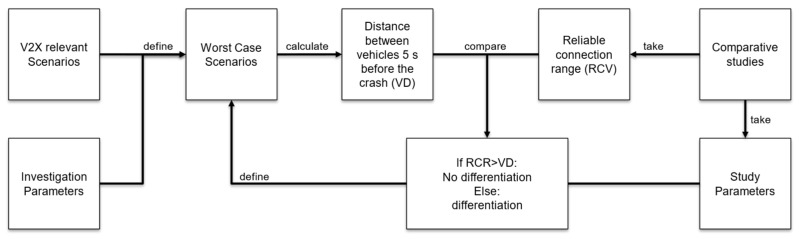
Schematic representation of the method used to determine the need for differentiation of V2X radio transmission technology.

Figure 1 schematically shows the methodological approach to answer the research question of whether V2X transmission technology needs to be distinguished within accident analysis in future studies. The method aims to calculate the distances of the vehicles 5 s before the crash and compare these with the reliable connection range from comparative studies. The comparison itself is carried out in the “Results and Discussion” section. The development of the scenarios and parameters investigated is described below.

Due to the practical-oriented working area of an accident analyst, future investigations will take place based on specific scenarios, which have already been determined in [18] according to their relevance for accident analysis and V2X.

To investigate the necessity to differentiate the radio transmission technologies, the performance differences of the competing V2X transmission technologies will be investigated in the relevant V2X accident scenarios according to [18]. Based on the three most relevant scenarios according to [18], it is determined by calculation whether the transmission technology within the accident scenario has to be considered from the point of view of the reconstruction of an accident. This estimates the relevance of the future transmission technology of V2X communication within the accident analysis.

Since many performance studies still refer to IEEE 802.11p and C-V2X LTE and further developments promise performance improvements [16], the more critical state-of-the-art IEEE 802.11p and C-V2X LTE are used for this investigation. Within LTE-V2X, there are also two different modes (mode 3 and mode 4) for sidelink communication [7]. This research focuses on 3GPP Rel-14 LTE-V2X PC5 mode 4, as it works without base stations and is, therefore, more comparable to DSRC [7]. A good comparison of the technical differences within the physical (PHY) and medium access (MAC) layer between DSRC and C-V2X can be found in [12].

The comparison of the two technologies will be made at the system level, as only the application-related differences are of interest to the accident analyst.

**Figure 2:**
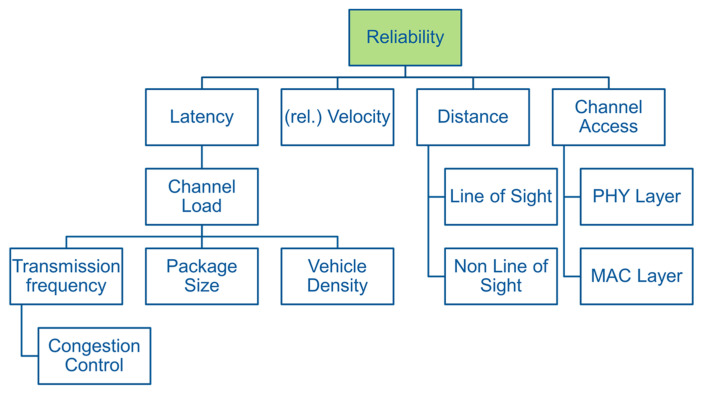
Performance parameters and their influencing factors with associated key performance indicators.

The basic requirements for the system performance of V2X communication with regard to safety-critical applications are high reliability, low latency times and high data throughput [16]. Reliability and latency are interdependent as can be seen in Figure 2. Since the data size of the messages sent for safety-critical applications is small compared to, for example, video streaming (average size of Cooperative Awareness Message (CAM) is 300 bytes [12]), the data throughput is of secondary importance when comparing the technologies.

The most important performance parameter is the reliability of data transmission and is measured with the key performance indicator (KPI) Packet Delivery or Reception Ratio (PDR/PRR). The PDR indicates how many of the sender’s message packets arrive successfully at the receiver [15]. The influencing factors here are the latency, the relative speed, the distance of the communication partners to each other and the channel access technologies. On the physical level, the multiplexing and coding procedures as well as the available bandwidth are particularly important [8]. For low latency times, fast access to the transmission channel is realised within the MAC layer [8]. C-V2X and DSRC use different approaches and methods, leading to differences in reliability and latency performance. The possible transmission and reception range is again dependent on the environment. If there are objects (houses, trucks, etc.) between the direct straight connection of the two vehicles, this is referred to as Non-Line of Sight (NLOS), which leads to a reduction in the transmission range compared to undisturbed connections (Line of Sight (LOS)). On the other hand, the latency time is mainly dependent on the channel load and is measured using end-to-end latency. The channel load is influenced by the number of vehicles (vehicle density), the transmission frequency and the transmitted packets’ size. The transmission frequency can be adapted to the prevailing channel load using Decentralised Congestion Control to avoid congestion [12].

Due to the many influencing parameters and the described but not presented mutual influences of the parameters, comparing the two technologies is only possible based on concretely defined boundary conditions. For the comparison, the reliability of data transmission is examined, since this also includes the influence of latency. The relevant literature defines reliability as a PDR > 90% over the transmission distance [15].

In the following, the three most critical different scenarios, frontal accident (50 + 51), rear-end accident (20–23) and turning accident (82 + 83), are investigated according to [18] under different environmental and boundary conditions forming the “worst case” scenarios from a V2X perspective. Among all turning scenarios, which are more critical than the rear-end collision scenario, the most critical scenario (82 + 83) was chosen as a representative. Table 1 lists the investigation parameters according to Figure 2 and how they were determined.

The most critical parameter in terms of performance is the distance between the C-ITS, as the signal strength decreases over the distance and is, therefore, decisive for the reliability of the data transmission. In addition, the distance is a constantly changing parameter within the V2X network and is of particular interest. The performance at system level is thus usually plotted as PDR over distance. Changes due to different speeds, NLOS, etc., can also be shown in this diagram. The distance is also of primary interest for the accident analyst, as it is necessary to reconstruct whether and from which time two C-ITS involved in a traffic accident could have communicated within the accident sequence. This question also represents the method for assessing the transmission technique. Thus, for each scenario, the distance $s_{D}$ to the collision point is calculated according to Equation (1) [19], assuming initial speeds $v_{0}$ of up to 60 km/h for urban regions, up to 100 km/h for rural roads and up to 140 km/h for motorways, which also correspond to the speeds of the comparison studies [8,9,10,12,15] in the results section. Deceleration is taken into account by $a_{D}$.

(1) $$s_{D}=\int_{t}^{\;}\left(v_{0}+a_{D}\times t\right)dt$$

**Figure 3:**
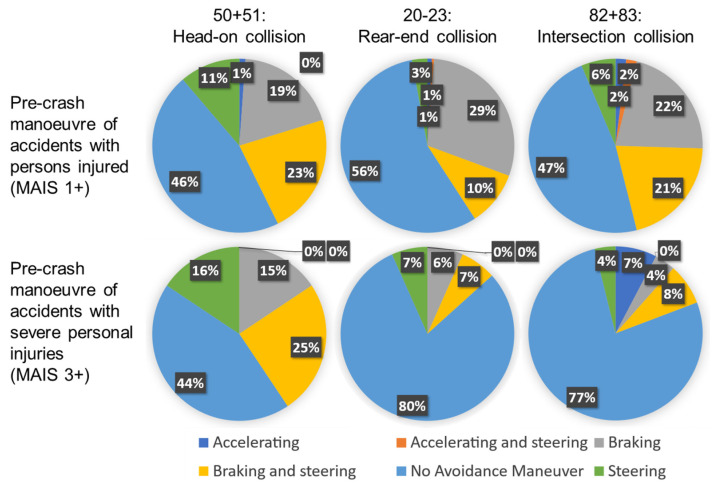
Manoeuvres of the drivers in the pre-crash within the selected scenarios divided into accidents with casualties, minor injuries and serious injuries.

Five seconds is the considered time t for the distance calculation within the scenarios, since the EDR according to UN R160 stores data for the reconstruction of the accident scene up to five seconds before the accident [20]. Thus, it is examined whether the vehicles are in contact over the entire accident scene to be reconstructed (5 s). No deceleration of the vehicles is assumed, as an evaluation of the Crash Investigation Sampling System (CISS) database of the NHTSA (General Vehicle datasets from 2017 until 2019) showed no reaction of the drivers in the scenarios in the majority of cases, which can be seen in Figure 3; here, especially in the serious rear-end and crossing accidents, we show that in 4 out of 5 cases there was no reaction. For approximately half of the accidents, this also applies to head-on collisions. In all diagrams, the percentage of braking reactions is smaller than the percentage of no reactions.

If due to the driving situation (e.g., a turn) a deceleration would have to be assumed to calculate the distance, an average speed is formed instead to determine the distance. This approach thus explains the different speed ranges given for each environment according to Table 1.

**Figure 4:**
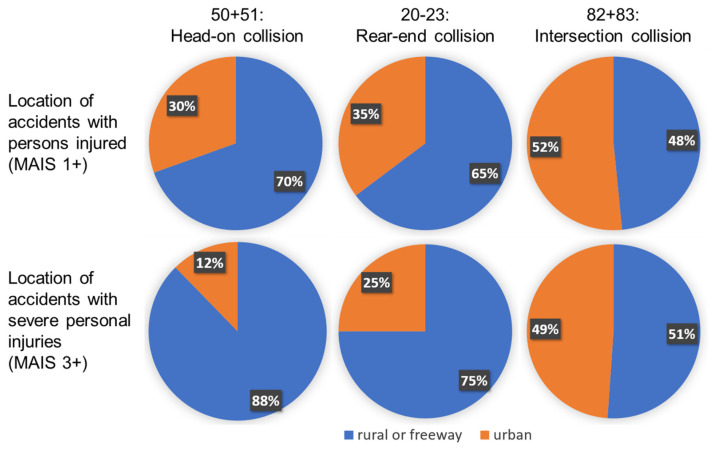
Locations of vehicle-to-vehicle accidents with slightly and seriously injured persons within the investigated scenarios.

The other investigation parameters are determined individually for each scenario. The possible environments are examined for each scenario, and the “worst-case” parameters are assumed. Figure 4 shows the results of the local assignment of the vehicle-to-vehicle accidents with injured persons as well as slightly and seriously injured persons per scenario. Since there is no direct locality parameter within the CISS, the locality was determined via the SPEEDLIMIT parameter. However, it is problematic that no uniform speed limits apply within the USA; therefore, it was impossible to differentiate between rural roads and freeways. However, the boundary between urban and rural and freeway could be set at 56 km/h (35 mph), as this was the maximum permissible speed in over 90% of the survey areas [21]. It should be noted that a sharp separation is not possible here either, but it is sufficient for assessing the accidents’ locality in this study’s context.

According to Figure 4, the oncoming traffic scenario, which can lead to a head-on collision (50 + 51), occurs most frequently outside built-up areas. Due to the demarcation of the driving directions on freeways, it can also be stated that accidents occur mostly on rural roads without demarcation. Accidents resulting in rear-end crashes also occur mainly outside built-up areas for minor and serious injuries. For serious accidents, the proportion of out-of-town accidents is even increasing. Differently from the oncoming crash scenarios, it is impossible to differentiate between rural roads and freeways. Therefore, the location for these scenarios is chosen in combination with the other parameters. Finally, it can be seen that intersection accidents (82 + 83) occur approximately 50% in built-up areas and 50% outside built-up areas. Due to the structural separation on motorways, it can also be concluded that out-of-town accidents occur on rural roads. Also, the choice of location is chosen together with the further investigation parameters to generate logical and, at the same time, critical scenarios.

**Figure 5:**
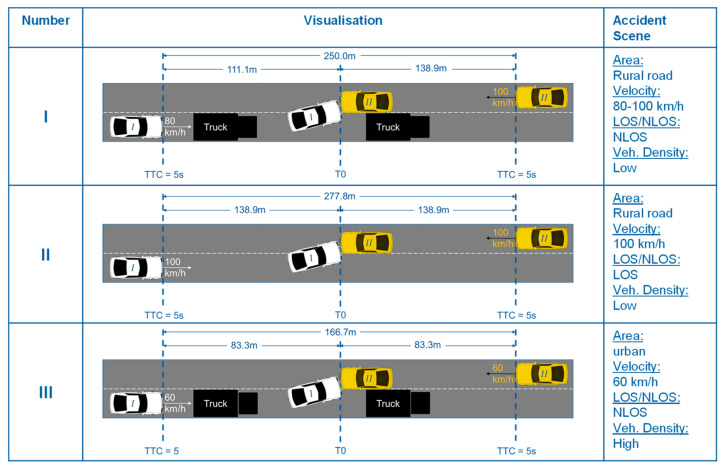
Illustration of the investigated “worst-case” oncoming traffic scenarios (50 + 51).

With the above-mentioned boundary conditions, scenarios I, II and III are shown in Figure 5. Scenario I describes a car following a truck on a rural road at 80 km/h corresponding to an NLOS connection to an oncoming vehicle (yellow). As there is no high traffic density on rural roads compared to urban areas and freeways, the traffic density is modelled as low.

In the LOS scenario II, the differential speed and thus the distance according to Equation (1) is greater for 5 s between the vehicles, so this is examined separately. Due to the higher traffic density in cities compared to rural roads, scenario III should also be investigated in urban areas. For the “worst-case” consideration, a truck is again modelled as an obstacle for an NLOS scenario. Since the vehicles were modelled at 60 km/h in urban areas in the studies used, this speed is also used in the urban scenarios for comparability.

**Figure 6:**
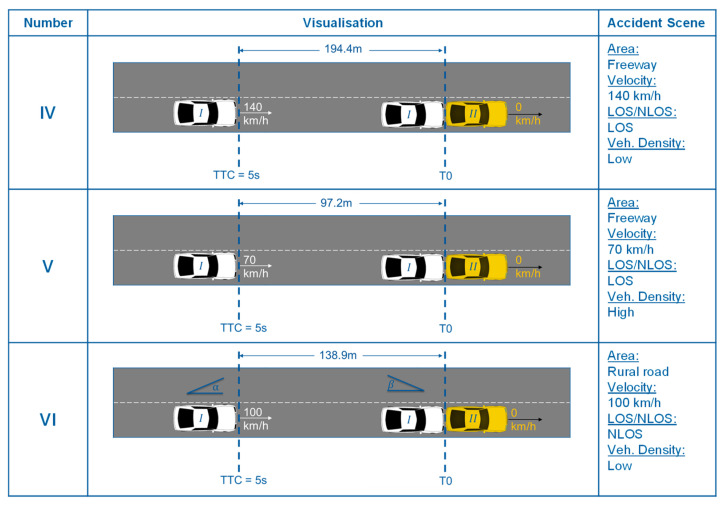
Illustration of the investigated “worst-case” rear-end crash scenarios (20–23).

The freeway or rural roads can be modelled for the rear-end crash scenarios for the surroundings. Considering the “worst-case” idea, a scenario on the freeway also includes the possible “worst-case” parameters of the scenarios on rural roads and in the city, as the speed and thus the distance is greatest on the freeway. At the same time, it is plausible to model a high traffic density. However, the modelling of an NLOS scenario does not make sense on the freeway concerning a rear-end crash, as there cannot be another vehicle between the crash partners that would generate an NLOS scenario. Also, an NLOS scenario is unlikely on the freeway within the distances investigated for infrastructural reasons. Therefore, two freeway scenarios with LOS and one rural road scenario with NLOS are investigated. Due to the speeds and the associated maximum distances, IV results in a scenario with low traffic density but high distance on the freeway. In reality, this scenario can correspond to an unbraked collision with the end of a traffic jam. Scenario V corresponds to a scenario with traffic already congested on the freeway, which is why only a speed of 70 km/h is modelled. On the other hand, a high traffic density can be plausibly taken into account for this scenario. Both scenarios can be seen in Figure 6. Also, a rural road scenario (VI) with infrastructure-related NLOS (e.g., a hilltop) is modelled. As rural roads do not have a high traffic density compared to cities and freeways, the traffic density is modelled as low.

**Figure 7:**
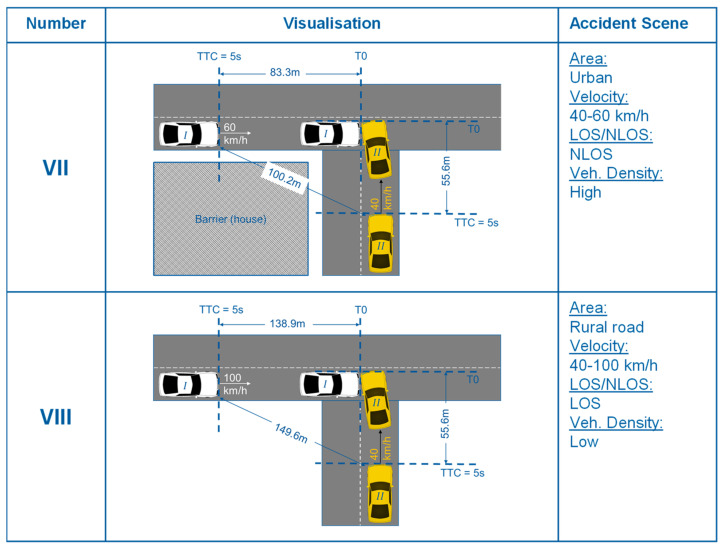
Illustration of the investigated “worst-case” turning crash scenarios (82 + 83).

A similar restriction for the oncoming traffic scenarios can be taken for the turning scenarios. Thus, no turning and crossing of the opposite lane should be possible on a freeway. Rural roads and urban roads are, therefore, relevant for the study of the crossing scenarios. Figure 7 shows the “worst-case” scenarios to be investigated. Scenario VII represents an NLOS intersection scenario in built-up areas. Both vehicles drive towards the intersection at 60 km/h. The turning process of the two vehicles leads to a collision. Due to the turning process of vehicle II and the associated deceleration, an average speed of 40 km/h is modelled. The traffic density is assumed to be high. In addition, a LOS turning scenario on a rural road with higher speeds and thus larger distances is investigated in VIII. The turning vehicle II is also modelled with an average speed of 40 km/h. The traffic density is assumed to be low.

## 3. Results and Discussion

In the following, the reliability between IEEE 802.11p and LTE-V2X PC5 Mode 4 is investigated for each scenario (I–VIII) using the relevant comparative studies from the literature. The studies investigating the state-of-the-art of transmission technologies at the system level are [8,9,10,12,15].

### 3.1. Oncoming Traffic Scenarios 50 + 51

In none of the relevant literature mentioned has scenario I been investigated exactly as described. However, similar boundary conditions can be found in the 5G Automotive Association (5GAA) tests, except for the differential speed [15]. Thus, the reliability of data transmission from a vehicle standing behind a truck (NLOS) to a vehicle driving in a loop at 32 km/h was tested there. As a result, 90% reliable data transmission is possible with DSRC up to a range of approx. 430 m. C-V2X achieves a range of 640 to 720 m [15]. It shows that both technologies should already have a reliable connection between the vehicles 250 m away 5 s before the crash. Despite the differences in speed between scenario I and the 5GAA trials, the range for both technologies should be sufficient to ensure a reliable connection between the vehicles. Thus, no distinction in transmission technology is necessary for future investigations related to accident analysis for this scenario.

Scenario II is also to be evaluated using the 5GAA trials considering the lower speed difference. In the equivalent test to that described above but without trucks (LOS), reliable ranges of 930 m for DSRC and 1350 m for C-V2X were measured. As described above, it can be assumed that at higher differential speeds a range of at least 277.8 m can also be expected according to the scenario. Reliable transmission should thus be realised within the area of interest by both technologies.

The last scenario, scenario III, has also not been investigated exactly in the literature under the mentioned boundary conditions. In [10,12], urban scenarios with NLOS due to buildings have been considered. According to [10], DSRC achieves a reliable data transmission of approx. 60 m and C-V2X of approx. 80 m. In [12], the reliable range is reported even more critically as approx. 25.1 m for DSRC and 31.6 m for C-V2X. The basic modelling of the scenario is identical for both sources. However, Ref. [12] models 1476 vehicles compared to [10] with only 590 vehicles in the relevant area, which explains the significant differences in the results. This shows that at a high vehicle density both technologies would not be able to realise a reliable data connection over the entire relevant distance of 166.7 m. However, it should be noted that buildings are used instead of a truck for the NLOS scenario in both modellings. The shielding by the buildings is much greater than would be expected from a truck. In addition, the modelled vehicle density is very high, especially in [12]. NLOS intersection scenarios were also investigated in the 5GAA studies. Here, a vehicle was parked between 2 trucks, and another vehicle drove by it at 32 km/h. In this case, a reliable data transfer is required for DSRC. A reliable data transmission of up to 400 m was determined for DSRC and 800 m for C-V2X. The range is reduced again by a higher differential speed and traffic density. Therefore, it can be stated that for this scenario, no clear statement on reliable transmission is possible based on the literature found.

### 3.2. Rear-End Crash Scenarios (20–23)

The Freeway scenarios IV and V can be found identically in [9,10]. In both cases, reliable transmission is simulated at 140 and 70 km/h at different traffic densities on a three-lane freeway in each direction. According to [9], a reliable range of 350 m is obtained for DSRC and 450 m for C-V2X at a speed of 140 km/h and a vehicle density of 60 vehicles per kilometre according to scenario IV. The vehicle density here corresponds to a 2.5 s distance and is thus 38.9% greater than the required minimum distance of 70 m (half speedometer). The same boundary conditions were set by [10]. However, the reliable range for DSRC is approximately 160 m, and C-V2X is approximately 290 m. Thus, in both studies, a connection using C-V2X should be given over the total distance of 194.4 m. According to [9], this is also true for DSRC, but the reliable range in [10] is insufficient for the total distance. The difference in the performance of these two studies can be attributed, in particular, to the simulated messages. Ref. [9] uses the real CAM generation model according to the European ETSI standard. In [10], on the other hand, constant message sizes are used with speed-dependent transmission rates. Thus, a transmission frequency of 10 Hz at 140 km/h has been modelled, leading to a higher channel load and thus to a lower reliable range than [9]. Since [9] represents the more realistic scenario, it can be assumed that both technologies have a connection over the distance to be reconstructed for scenario IV.

Scenario V has also been simulated by [9,10] in the same way. For 70 km/h, a distance of 2.5 s results in a traffic density of 120 vehicles per kilometre. According to [9], with the ETSI CAM generation model, the range is 300 m for DSRC and 350 m for C-V2X. According to [10], the reliable ranges are 170 m for DSRC and 270 m for C-V2X. According to both investigations, a reliable connection is guaranteed within the 97.2 m distance of the scenario with both technologies.

A concrete investigation of scenario VI has not been done in the literature. However, the NLOS investigations of the 5GAA with lower speed can be considered approximate. As described in scenario III, reliable ranges of 400 m and 800 m were determined for DSRC and C-V2X, respectively. Therefore, despite higher speeds, it can be assumed that a reliable connection exists within 138.9 m regardless of the transmission technology.

### 3.3. Turning Scenarios (68 + 69)

Scenario VII is examined by the studies described for scenario III [10,12] with similar boundary conditions. Unlike scenario III, the shielding in scenario VII is provided by buildings as in the studies. Therefore, the reliable ranges should correspond to the two studies depending on the traffic density. For large traffic densities, the reliable ranges of 25.1 m to 60 m for DSRC and 31.6 m to 80 m for C-V2X are not sufficient for the entire accident scene of 98.1 m. Since the traffic density in both studies is very high, it is, in principle, possible for both technologies to establish a connection over this distance as shown by the 5GAA study also described in scenario III. As in scenario III, whether the transmission technology for accident analysis studies must be differentiated in the future cannot be answered clearly. In principle, it can be seen that C-V2X has a higher range than DSRC. However, both are not large enough to cover the total distance to be reconstructed.

Lastly, the 5GAA study can again be used for LOS investigations for turn scenario VIII, as no other studies have investigated rural roads. With a reliable range of 930 m for DSRC and 1350 m for C-V2X, a reliable connection over 155.3 m should be realised by both technologies even at higher speeds as described in scenario VIII.

### 3.4. Summary

Finally, Table 2 summarises the results. The necessary distances for the accident reconstruction are entered in the “Scenarios” column. The columns next to it show the literature results examined. If the field has no pattern, the respective scenario strongly agrees with the respective study. If the field is dotted, minor deviations must be considered, such as a different speed or vehicle densities. A shaded field means that the study does not cover the scenario. The last “Conclusion” column shows the results from the above discussion of the individual scenarios. If the field is green, there is no need to distinguish the transmission technology in future studies on this scenario. If, on the other hand, the field is red, there is a significant difference in performance between the technologies that would lead to different results in the accident reconstruction and must, therefore, be taken into account. However, this is not a certainty for any of the scenarios.

On the other hand, the urban scenarios III and VII represent borderline cases, as both transmission technologies cannot provide a sufficiently large reliable distance for accident reconstruction at high traffic densities. A distinction could thus be necessary at high traffic densities, as C-V2X has a higher range than DSRC, and vehicles can thus be in contact approx. 6 to 20 m earlier. At a relative speed of 120 km/h for scenario III, this results in an earlier reliable connection of 0.18 to 0.6 s and at 72 km/h for scenario VII, 0.3 to 1.0 s. However, the 5GAA trials show that a reliable connection over the relevant distance is possible for both technologies at a low traffic density. Thus, it depends on the traffic density of the study in urban scenarios whether the transmission technology needs to be differentiated for the area of interest.

## 4. Conclusions

The results show that investigations on freeways and rural roads can be carried out independently of the transmission technology and the other existing boundary conditions (speed, traffic density, NLOS/LOS). This is not the case for studies in urban areas, where both technologies may not have a sufficiently reliable connection range depending on the traffic density.

Furthermore, it should be noted that most studies deal with freeways and urban environments. Specific studies on rural roads are not to be found, as these have probably been classified as uncritical due to the low traffic density and thus channel load. If this is the case, this should be determined in new studies. In addition, it can be seen that the studies directly cover only three out of eight of the “worst-case” scenarios. For future studies, the scenarios shown here can thus serve as a reference point for a larger variance of the study area. Also, more studies should be made with real vehicles to validate the results of the many simulations.

The results from all studies show that C-V2X has a higher reliable range than DSRC in all scenarios and under all boundary conditions. Nevertheless, a differentiation of the transmission technology within accident analysis is probably not necessary today nor will it be in the future due to the further development of the transmission technologies to WLAN bd and NR-V2X. As described in [16], both developments aim to increase the reliability range. At the same time, reliable transmission with lower latency at higher relative speeds and traffic densities should be possible. Initial performance studies [3,22,23] already show great progress in this development. Thus, in the future, reliable transmission within the required distance of the scenarios investigated should also be possible in urban areas.

## Figures and Tables

**Table 1:** Determination of the study parameters.

Study Parameters	Value
Distance	Kinematics according to boundary conditions by environment and reconstruction period (5 s EDR)
Velocity	urban: 40–60 km/h rural: 80–100 km/h freeway: 70–140 km/h
Channel Access	According to the referenced studies
LOS/NLOS	NLOS as “worst case” For higher speed differences also LOS
Transmission frequency/Package size	According to the referenced studies ~CAM (10 Hz/~300 Byte)
Vehicle density	In general: Urban and freeway: high density Rural: low density

**Table 2:**
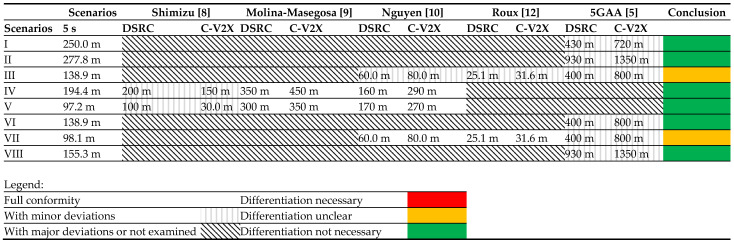
Summary of the calculated ranges of the scenarios and the corresponding studies from the literature.

## Data Availability

Publicly available datasets were analysed in this study. These data can be found here: https://www.nhtsa.gov/file-downloads?p=nhtsa/downloads/CISS/2017/ (accessed on 15 March 2022), https://www.nhtsa.gov/file-downloads?p=nhtsa/downloads/CISS/2018/ (accessed on 15 March 2022) and https://www.nhtsa.gov/file-downloads?p=nhtsa/downloads/CISS/2019/ (accessed on 15 March 2022).
